# Preparation of Fluorescently Labeled Chitosan-Quercetin Drug-Loaded Nanoparticles with Excellent Antibacterial Properties

**DOI:** 10.3390/jfb13030141

**Published:** 2022-09-04

**Authors:** Jingxin Zhou, Na Li, Ping Liu, Zhiwei Liu, Lili Gao, Tifeng Jiao

**Affiliations:** State Key Laboratory of Metastable Materials Science and Technology, Hebei Key Laboratory of Nanobiotechnology, Hebei Key Laboratory of Heavy Metal Deep-Remediation in Water and Resource Reuse, Yanshan University, Qinhuangdao 066004, China

**Keywords:** fluorescence labeling, nanoparticle, chitosan, fluorescein isothiocyanate

## Abstract

In recent years, quercetin plays an increasingly important role in the medical field. However, the absorption and effect of quercetin as a drug in vivo are limited due to its extremely poor solubility in water. In addition, chitosan nanoparticles can deliver poorly soluble drugs as drug delivery carriers. Herein, chitosan nanoparticles were prepared by oxidative degradation and ionic cross-linking technology to study the drug loading properties of quercetin. On the other hand, the application of chitosan for fluorescent materials can improve the biocompatibility of fluorescent materials and increase the adsorption of fluorescent materials. Fluorescently labeled chitosan nanoparticles, especially chitosan microsphere fluorescent probes prepared using the abundant amino groups on chitosan chains to react with fluorescein isothiocyanate (FTIC), have been widely used as fluorescent probes in biomarkers and medical diagnostics. Therefore, chitosan–quercetin (CS–QT) drug-loaded nanoparticles are labeled with FITC, and the drug-loaded rate, encapsulation efficiency, and antioxidant properties were investigated. The drug-loaded rate of the sample reaches 8.39%, the encapsulation rate reaches 83.65%, and exhibits good antioxidant capacity. The fluorescence aperture of the obtained sample was consistent with the inhibition zone, which could realize the visualization of the antibacterial performance of the sample. The fluorescent-labeled nano-system exhibit superior antibacterial properties, which provide a strategy for observing the release and function of drugs.

## 1. Introduction

Nowadays, the use of natural biopolymers in science is on the rise [1]. As is known, chitosan is widely applied in the field of biological clinical medicine due to its biological properties, stable chemical properties, and non-toxicity [2]. The superiority of chitosan not only has various properties such as adhesion, antibacterial and antioxidant, but also exhibits great advantages in the transport of substances [3]. In addition, biomedicine, cosmetics, and other industries require chitosan with a molecular weight of less than 100,000, which is used for sustained release. The chitosan used as a carrier is mostly used in the form of nano/microspheres [4]. For example, Taís Gratieri et al. evaluated the potential of an in situ gel-forming delivery system comprised of poloxamer/chitosan as well as a chitosan solution as vehicles for enhanced permeation and sustained release of drugs [5]. Both the gel and the chitosan solution exhibited an enhanced permeation of fluconazole, in comparison with aqueous solution. Nuran Işıklan et al. developed stable temperature-responsive chitosan/hydroxypropyl cellulose (CS/HPC) blend nanospheres that are able to deliver the drug to the small intestine. The temperature-responsive CS/HPC blend nanospheres open up exciting avenues for biomedical applications [6]. Abdurrahim et al. prepared calcium/chitosan nanoparticles (Ca/CS NPs) and investigated their potential as a new method for preserving paper documents. Antifungal and antibacterial assays showed that calcium decoration increased the antibacterial activity of nanoparticles by reducing the required dose and increasing the inhibitory effect. This antimicrobial activity also stabilizes the pH of the paper product [7]. Wang et al. developed chitosan nanoparticles as a new controlled release system for natural biocides. Then, the antimicrobial activities of thymol-loaded chitosan nanoparticles against microorganisms isolated from the Feilaifeng limestone’s microbial community were studied, together with the kinetics and mechanism of thymol released from chitosan nanoparticles in water [8]. Shinde et al. synthesized the water-soluble chitosan derivative N-trimethyl chitosan (TMC) and prepared flurbiprofen (FLU): hydroxypropyl-β-cyclodextrin (HP-β-CD) composite loaded nanoparticles for the treatment of bacterial conjunctivitis. The developed TMC nanoparticles provided extended-release potential for transmucosal ocular delivery of hydrophobic flurbiprofen [9]. Zhao et al. prepared DNA vaccines encapsulating chitosan nanoparticles by a complex coalescence method to enhance the efficacy of DNA vaccines against swine influenza. Chitosan nanoparticles containing a conjugated DNA vaccine against swine influenza were evaluated for triggering immune responses in BALB/c mice, laying the groundwork for future work on a broad range of gene delivery systems including DNA vaccines [10]. Ainali et al. prepared pure chitosan and its grafted derivatives of fluticasone propionate (FLU) and salmeterol cetaphate (SX) drugs for chronic obstructive pulmonary disease (COPD) by an ionic gel technique, improving their in vitro release properties and bioavailability [11].

As a carrier, chitosan nanospheres (CSNP) can be loaded with insoluble drugs, such as quercetin, which is 3,3’ 4’ 5,7-pentahydroxyflavone. Quercetin widely exists in natural crops such as vegetables, fruits and olive oil [12]. It is easily soluble in organic solvents such as chloroform and ethanol, but the solubility in water is extremely poor. Therefore, the absorption and function of quercetin as a drug are limited in organisms. Quercetin plays an important role in the medical field, including anti-tumor, anti-oxidation and cerebral vascular protection. For example, Smith et al. synthesized and evaluated four new cocrystals of quercetin (QUE): quercetin:caffeine (QUECAF), quercetin:caffeine:methanol (QUECAF·MeOH), quercetin:isonicotinamide (QUEINM), and quercetin:theobromine dihydrate (QUETBR·2H_2_O). Compared with QUE alone, the water solubility of the four cocrystals was improved to varying degrees. The results of this study further implicate the potential for co-crystallization in drug development [13]. Fan et al. showed that quercetin may be a promising tyrosinase inhibitor and may have a potential application as a dietary supplement for the treatment of pigmentation disorders [14]. Xu et al. concluded that quercetin is effective in the treatment and prevention of human diseases since it influences glutathione, enzymes, signal transduction pathways, and reactive oxygen species (ROS) production [15]. Kyuichi et al. introduced the effects of quercetin and its related polyphenols on the brain, blood vessels, muscles and intestines, emphasizing that the roles of quercetin and its related polyphenols in preventing neurodegenerative diseases, mood disorders, atherosclerosis and metabolic syndrome and other diseases have certain potential [16,17]. Tang et al. summarized the evidence for the pharmacological potential and inhibition of quercetin on cancers. A large number of in vivo and in vitro experiments have shown that quercetin has a strong role in promoting apoptosis, inhibiting metastasis, and regulating cell cycle and tumor angiogenesis [18]. Andrea et al. concluded that quercetin can also be used in the treatment of diabetes/obesity and circulatory dysfunction, including inflammatory and mood disorders. In addition, drug metabolism and major drug interactions, as well as potential toxicity, will be also spotlighted [19]. Li et al. used maize alcohol soluble protein/soluble soybean polysaccharide (SSPS) nanoparticles to encapsulate hydrophobic quercetin, resulting in the significantly enhanced photochemical stability and scavenging ability of quercetin. This study shows that these composite nanoparticles can be used as an all-natural delivery system for bioactive molecules in food and pharmaceutical preparations [20]. Chitosan is the only alkaline polysaccharide in nature, with good biocompatibility, non-toxicity, and is degradable to organisms. Wang et al. synthesized a new type of amphiphilic chitosan (ACS) with deoxycholic acid (DA) as the hydrophobic group and N-acetyl-L-cysteine (NAC) as the hydrophilic group. Quercetin was encapsulated by ultrasonic self-assembly to prepare amphiphilic chitosan quercetin nanomicelles (ACS-QNMs). Studies have shown that after quercetin is encapsulated by amphoteric chitosan, it can be slowly released at the human body temperature of 37 °C and stored stably at room temperature [21]. Aluani et al. found that chitosan/sodium alginate as antioxidant activity of carrier-loaded quercetin was enhanced, and quercetin nanoparticles had no significant cytotoxicity in vitro [22]. Therefore, we believe that combining biocompatibility and improved protective activity of encapsulated antioxidants, chitosan nanoparticles can be considered quercetin suitable carriers.

Fluorescein isothiocyanate (FITC) is widely used in fluorescent labeling with enhanced quantum yield, stable optical properties and good biological properties [23]. For example, Tatiana et al. proposed a new approach for visualization of the intracellular distribution of triterpene acids, based on fluorescent labeling by FITC. Experimental tracing of the dynamics of penetration and distribution of the labeled ursolic acid has shown that when the acid enters the cell, it initially localizes on the inner membranes where the predicted target Akt1/protein kinase B is located [24].

**Figure 1:**
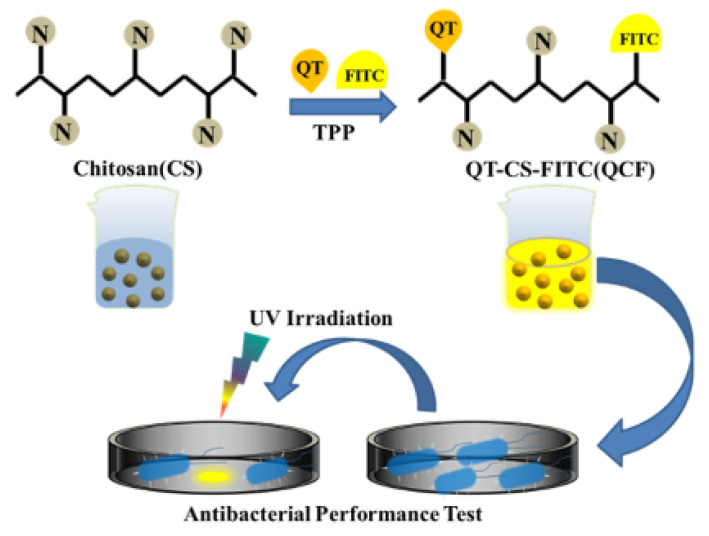
Schematic diagram of the experimental process.

In this work, we prepared CSNPs following previous work and used them to study the drug-loaded performance of quercetin, optimized the performance with drug-loaded rate and encapsulation rate, and explored its antibacterial performance [25]. The schematic diagram of the experimental principle was shown in Figure 1. It was proved that the fluorescein was successfully labeled and that the nanomedicine had a certain inhibitory effect on *Escherichia coli* through structural characterization, spectral characterization, and other means.

## 2. Materials and Methods

### 2.1. Materials

Chitosan (CS, deacetylation degree ≥95%), hydrochloric acid (HCl), sodium hydroxide (NaOH), isothiocyanate, fluorescein, acetic acid (HAc), hydrogen peroxide (H_2_O_2_), sodium tripolyphosphate (TPP), dimethyl sulfoxide (DMSO), quercetin (QT), sodium acetate trihydrate (C_2_H_3_O_2_Na·3H_2_O), sodium bicarbonate (NaHCO_3_), sodium carbonate (Na_2_CO_3_), dibasic hydrogen phosphate Sodium dihydrate (Na_2_HPO_4_·2H_2_O), sodium dihydrogen phosphate dihydrate (NaH_2_PO_4_·2H_2_O), ferrous sulfate heptahydrate (FeSO_4_·7H_2_O), salicylic acid (C_7_H_6_O_3_), pyrogallol (C_6_H_6_O_3_), tris (hydroxymethyl) aminomethane salt (C_4_H_12_ClNO_3_, Tris-HCl) and other medicines were purchased from Shanghai Aladdin Co., Ltd. (Shanghai, China). Absolute ethanol and agar were purchased from Sinopharm Chemical Reagent Co., Ltd. (Beijing, China). Yeast extract peptone was purchased from Beijing Obosing Biotechnology Co., Ltd. (Beijing, China). Deionized water was obtained using a Milli-Q ultrapure water purification system.

### 2.2. Preparation of Chitosan-Quercetin Drug-Loaded Nanoparticles

**Figure 2:**
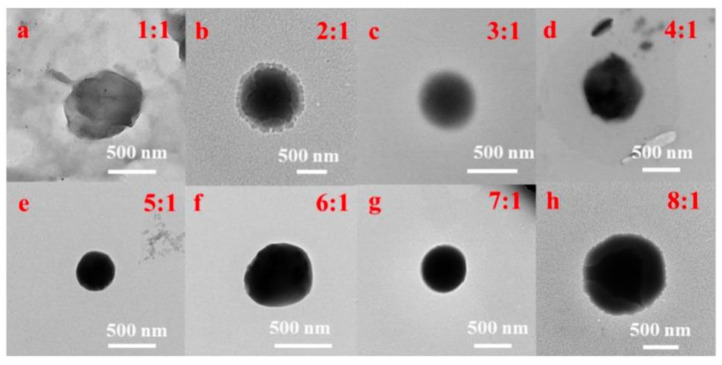
TEM images of QC with the volume ratios of (**a**–**h**) 1:1, 2:1, 3:1, 4:1, 5:1, 6:1, 7:1, and 1:1 of chitosan carrier to quercetin.

The CSNP was synthesized based on our previous work [25]. Then, appropriate quercetin–absolute ethanol solution was added into the CSNP-acetic acid solution to make uniform CS-QT drug-loaded nanoparticles. First, 10 mg of quercetin was added into absolute ethyl alcohol (10 mL), and the solution was mixed evenly to prepare 1 mg/mL of absolute ethyl alcohol solution of quercetin. Then, the solution was diluted to 100, 10, 5, 2.5 and 1 μg/mL with absolute ethanol to determine the standard solution curve. 160 mg of CSNP powder was dissolved in an acetic acid solution (80 mL, 1%), and the pH value was adjusted to 5.5 with 5 mol/L of NaOH solution. The 2 mg/mL chitosan solution was mixed with 1 mg/mL of quercetin–absolute ethanol solution in the volume ratio of 8:1, 7:1, 6:1, 5:1, 4:1, 3:1, 2:1, and 1:1, and the sodium tripolyphosphate (TPP) solution with the mass volume percent concentration of 0.2% was added for crosslinking while stirring under dark conditions. That is, the mass ratio of chitosan carrier to quercetin was 16:1, 14:1, 12:1, 10:1, 8:1, 6:1, 4:1, and 2:1. As shown in Figure 2, the TEM images showed that the drug-loaded nanoparticles with the mass ratio of chitosan carrier to quercetin of 10:1 (volume ratio of 5:1) were the best. The drug-loaded microspheres were denoted as QC. Anhydrous ethanol solution of quercetin (1 mg/mL) was prepared and diluted with solvent to 1, 5, 10, 25, 50, 75, and 100 μg/mL, respectively, and the standard solution curve was determined by testing the UV absorption value. The ultraviolet absorption spectrum of quercetin anhydrous ethanol solution is at 374 nm, which can be used as the basis for the content determination.

### 2.3. Preparation of Fluorescence-Labeled Chitosan-Quercetin Drug-Loaded Nanoparticles

First, 1 mL of quercetin–ethanol solution, fluorescein isothiocyanate-dimethyl sulfoxide solution (200 μL, 10 mg/mL) and 1.66 mL of TPP solution with a mass volume concentration of 0.2% were added to the chitosan–acetic acid solution (5 mL, 2 mg/mL), and the reaction was carried out the dark conditions for 3 h under magnetic stirring. The complex of quercetin–chitosan–fluorescein isothiocyanate (QT-CS-FITC) was denoted as QCF.

### 2.4. The Rate of Drug-Loaded and Encapsulation Testing

The chitosan carrier and quercetin were mixed in the ratio of 8:1, 7:1, 6:1, 5:1, 4:1, 3:1, 2:1, and 1:1, respectively, under light-proof conditions, and TPP solution of 0.2% by mass was added while stirring, and then the absorbance of the supernatant was measured by centrifugation, the volume of the supernatant was measured to calculate the content of quercetin in the supernatant. The material was weighed three times after centrifugation, washing and precipitation of the dried material

### 2.5. Antioxidant Performance of QC

The clearance rate test of O_2_-·: First, HCl solution, Tris-HCl solution, and o-triphenol solution were prepared at concentrations of 8 mmol/L, 50 mmol/L, and 3.5 mmol/L, respectively. Next, 0, 0.1, 0.2, 0.3, 0.4, 0.5, 0.6 mL of 0.5 mg/mL of QC solution were added to 3.5 mL of Tris-HCl solution, respectively, then samples were filled up to a 4.0 mL final volume with ultrapure water and mixed evenly. After heating at 37 °C for 10 min, 0.5 mL of 3.5 mmol/L o-triphenol solution was added to the solution. The reaction of the control group without QC solution and the other six experimental groups was carried out for 6 min under the same hydrothermal conditions, and the reaction was stopped after adding 0.5 mL of HCl solution rapidly. Then, the UV absorption values at 300 nm were tested after the experiment was completed.

The clearance rate test of OH·: First, H_2_O_2_ solution, FeSO_4_ solution and salicylic acid-anhydrous ethanol solution (SA) were prepared at concentrations of 0.1%, 6 mmol/L and 6 mmol/L, respectively. Then, 2 mL of different concentrations of QC solution were measured and 1 mL each of FeSO_4_ solution, SA solution, and H_2_O_2_ solution was added sequentially. Ultrapure water was added to make up the volume to 10 mL and then mixed well, then the samples were heated in 37 °C water bath for 30 min. The samples were replaced with 2 mL of anhydrous ethanol in the control group and 1 mL of 0.1% H_2_O_2_ solution was replaced with 1 mL of ultrapure water in the reference group. The clearance ability of OH· was investigated according to the change of UV absorption value at 510 nm.

### 2.6. Antimicrobial Resistance Testing

The experiments were performed under aseptic conditions. First, the sterilized Petri dishes and solid medium were preheated in a drying oven at 50 °C. The bacterial solution (1mL, and 1 mL of sterile distilled water for the negative control group) was gently poured into Petri dishes, and the Petri dishes immediately shaken gently to mix them well, ensuring that each has basically the same amount of the bacteria. The Oxford Cup method was further used to test the antimicrobial properties of QCF. The Oxford cup was a 6.0 mm high stainless steel tube with an outer diameter of 8.0 mm. The Oxford cup was then placed in the center of the Petri dish filled with solid medium, and 200 μL of the sample (200 μL of sterile distilled water for the negative control) was aspirated into the Oxford cup and incubated for 24 h at 37 °C.

### 2.7. Characterization

UV-vis spectra and fluorescence spectra of the liquid samples were obtained using a Shimadzu UV-2550 system (Shimadzu Corporation, Kyoto, Japan). Infrared spectra measured using a Fourier infrared spectrometer (Thermo Nicolet Corporation, Waltham, MA, USA) were used to analyze the structure and composition of the samples. Field emission scanning electron microscopy (FE-SEM) (S-4800II, Hitachi, Tokyo, Japan) and inverted fluorescence microscopy (TS100, Nikon Instruments (Shanghai) Co., Ltd., Shanghai, China) were used to observe the surface morphology of the samples.

## 3. Results and Discussion

### 3.1. Performance Analysis of Chitosan-Quercetin Drug-Loaded Microspheres (QC)

As shown in Table 1, the pH, supernatant UV absorbance (Ab), drug-loaded rate (DL) and encapsulation rate (EE) of the solution are calculated according to Equation (2), where W_1_ is the mass of supernatant quercetin, W_2_ is the sample mass after three times of centrifugation, washing, precipitation and drying, W_3_ is the mass of quercetin added in the preparation of nanoparticles.

DL = (W_3_ − W_1_)/W_2_ × 100% (1)

The drug-loaded rate and encapsulation rate are very important criteria to judge the drug-loaded performance. Under the present experimental conditions, the drug-loaded rate reached 8.39% and the encapsulation rate reached 83.65% when the volume ratio of chitosan carrier to quercetin was 5:1. Consequently, the system with a volume ratio of chitosan carrier: quercetin = 5:1 was selected as the optimized formulation to the drug-loaded performance of the particles.

The pyrogallol auto-oxidation is performed when it is in a weak alkaline environment, during which the O_2_^−^· and colored products are produced, and substances with antioxidant effect can inhibit the pyrogallol auto-oxidation [26,27]. Therefore, the UV absorption value is tested at 300 nm. According to pyrogallol autoxidation method, the scavenging rate of superoxide anion is tested by QC. The clearance rate of O_2_^−^· is calculated by Equation (3), where R_1_ is the clearance of O_2_^−^· by the QC complex, B_0_ is UV absorbance of the control groups at 300 nm, and B_1_ is UV absorbance of samples at 300 nm.

(2) $$R_{1}=\frac{B_{0}-B_{1}}{B_{0}}\times 100\%$$

**Figure 3:**
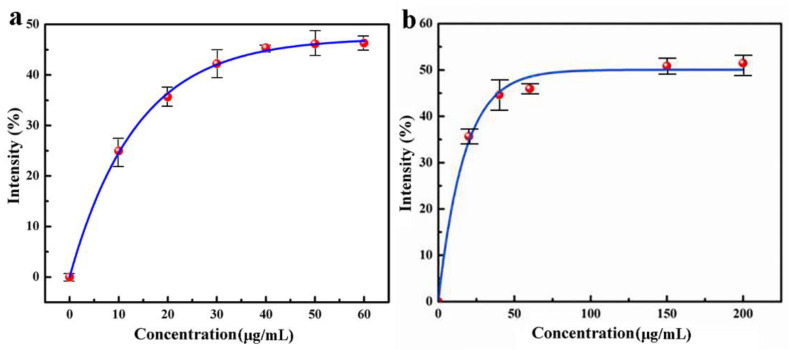
Antioxidant capacity analysis diagram of QC: (**a**) obtained by detecting the UV absorbance of the solution at 300 nm; and (**b**) obtained by detecting the UV absorbance of the solution at 510 nm.

The experimental results are shown in Figure 3a, indicating that the superoxide anion scavenging ability of QC increases with the increase of concentration. When the concentration of nanomedicine reaches 48.4 μg/L, the superoxide anion scavenging rate is 45.9%, but when the concentration of nanomedicine increases continuously, the superoxide anion clearance rate does not increase. Therefore, under the experimental condition, the superoxide anion scavenging rate of CS–QT nanomedicine can reach 45.9%.

The clearance rate of QC to OH· is tested based on Fenton reaction. The colored substances can be produced by salicylic acid and OH· with characteristic UV absorption at 510 nm. The antioxidant substances present in the solution react preferentially with OH· and the colored substances are reduced [28,29]. The OH· scavenging ability of the nanoparticles was investigated according to the change of UV absorption value at 510 nm. It is calculated by Equation (3), where R_2_ is the clearance of OH· by the QC complex, C_0_ is UV absorbance of the control group at 510 nm, C_1_ is UV absorbance of samples at 510 nm, and C_2_ is the UV absorbance of the reference group at 510 nm.

(3) $$R_{2}=\left[1-\frac{C_{1}-C_{2}}{C_{0}}\right]\times 100\%$$

Figure 3b was obtained by detecting the UV absorbance of the solution at 510 nm. The data shows that within a certain range, the hydroxyl radical scavenging ability of nanomedicine increases with the increase of concentration. When the concentration of nanomedicine reaches 70.7 μg/L, the hydroxyl radical scavenging rate is 49.2%, and then the scavenging rate does not increase continuously with the increase of nanomedicine concentration. Therefore, under the experimental condition, the highest scavenging rate of QC on the hydroxyl radical is 49.2%.

### 3.2. Performance Analysis of Quercetin-Chitosan-Fluorescein Isothiocyanate (QCF)

**Figure 4:**
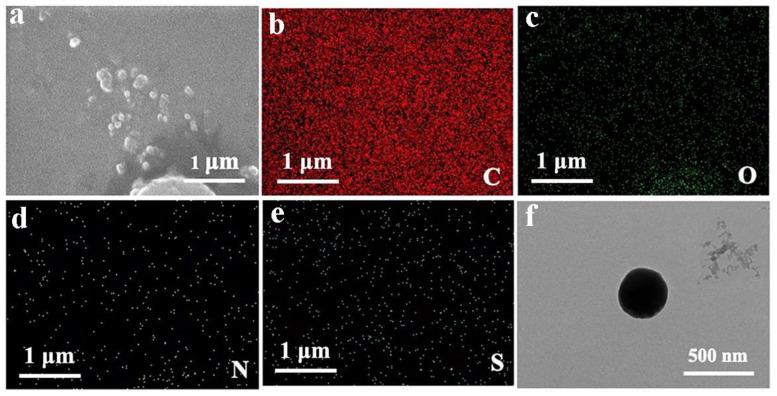
SEM image of QCF. (**a**) SEM image of QCF with (**b**–**e**) C/O/N/S/element mappings; and (**f**) TEM image of QCF.

As shown in Figure 4a–e, there are few particles in the SEM images, and most of the particles are agglomerated. Compared with chitosan alone, the element distribution diagram has an S element, which is a characteristic element of fluorescein. Therefore, SEM images can prove that fluorescein is successfully crosslinked with chitosan and quercetin. As shown in the TEM image of Figure 4f, the surface of the nanoparticles is regular and the shape is approximately spherical, and the experimental effect is great. The diameter of the particles is around 500 nm.

**Figure 5:**
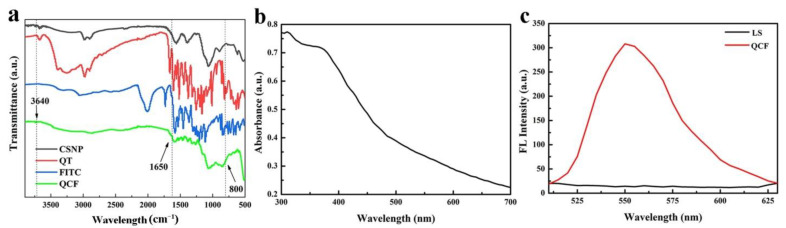
(**a**) FT-IR spectra of CSNP, quercetin, FITC and QCF; (**b**) UV-vis spectrum of QCF; and (**c**) fluorescence spectra of LS and QCF.

The infrared spectra of CSNP, quercetin, FITC and QCF are shown in Figure 5a. Compared with chitosan, the -OH and -NH stretching vibration peaks of QCF at 3640 cm^−1^ are weaker, and the -NH bending vibration at 1650 cm^−1^ is weaker. Compared with FITC, the characteristic peak at 800 cm^−1^ of fingerprint area is disappeared, which proves the successful crosslinking of fluorescein. In UV-vis spectrum (Figure 5b), QCF has a broad absorption at 350–400 nm, and the absorption peak is at 375 nm. The excitation wavelength of the fluorescence spectrum is set to 375 nm according to the absorbance of the ultraviolet-visible spectrum. The fluorescence spectra of supernatant (LS) after washing repeatedly and QCF are shown in Figure 5c. The fluorescence peak at 550 nm is the characteristic fluorescence emission peak of fluorescein. After washing repeatedly, the free fluorescein is washed. There is no characteristic emission peak at 550 nm, but the fluorescence peak of QCF is obvious, which proves that fluorescein is successfully cross-linked.

**Figure 6:**
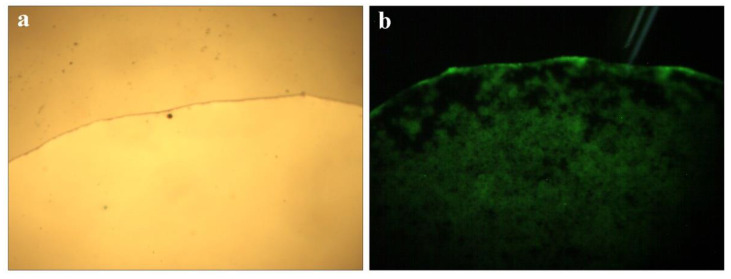
Microscope picture of QCF solution (**a**,**b**).

Microscope images of QCF solution are shown in Figure 6a,b under visible light and ultraviolet light. The solution emits blue and green light under the excitation of ultraviolet light, and the characteristics are shown by the fluorescence spectrum of Figure 5c, in which the peaks are consistent.

**Figure 7:**
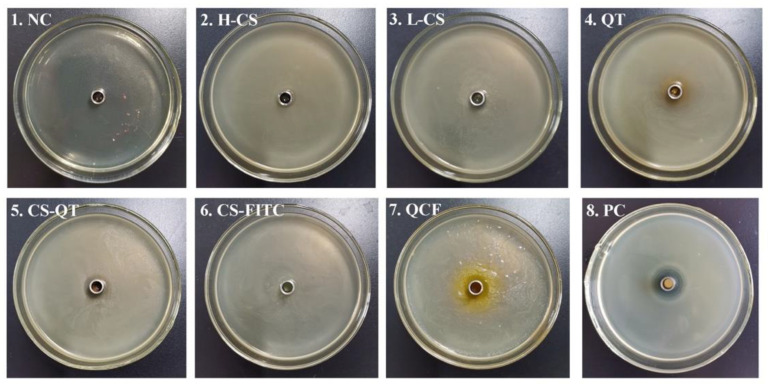
Diagram of *E. coli* culture. The medium of No. 1 to No. 8 is NaCl negative control (NC), high molecular weight chitosan (H-CS) solution, the prepared chitosan microsphere (L-CS) solution, quercetin (QT) solution, chitosan nanospheres–quercetin (CS-QT) solution, chitosan nanospheres–fluorescein isothiocyanate (CS-FITC) solution, quercetin–chitosan–fluorescein isothiocyanate (QCF) solution, and cephalexin positive control (PC), respectively.

Chitosan is widely used as an antibacterial agent because of its broad antimicrobial action and its non-toxicity and good biodegradability. A large number of chitosan derivatives with significant bacterial inhibitory effects have been widely reported [30,31]. It has been reported that low relative molecular mass chitosan and its derivatives exhibit good inhibitory activity against E. coli, fungi and yeasts [32,33]. For example, Li et al. loaded chitosan/xylosulfonate complexes onto cellulose surfaces by stack deposition and showed good inhibition activity against *E. coli* [34]. The Oxford cup method is a common method used to conduct bacteriostatic tests [35,36,37,38]. The Oxford cup method is used to test the ability of QCF to inhibit *E. coli* for the antibacterial properties of nanomedicine, the currently generally accepted mechanism is that chitosan will penetrate into microorganisms, and it will combine with DNA in bacterial cells to prevent mRNA and protein synthesis, causing metabolic disorders. Therefore, activities such as growth and reproduction will be restricted [39,40,41,42]. The antibacterial mechanism of quercetin is still under study, and the generally accepted mechanism is that quercetin can remove reactive oxygen species, promote electron transfer, regulate nuclear transcription factors, and inhibit bacterial growth and reproduction [43]. In this experiment, NaCl negative control (NC) and cephalexin positive control (PC) are set up. Test samples include high molecular weight chitosan (H-CS) solution, prepared chitosan microsphere (L-CS) solution, quercetin (QT) solution, chitosan nanospheres–fluorescein isothiocyanate (CS-FITC) solution, chitosan nanospheres–quercetin (CS-QT) solution, and quercetin–chitosan–fluorescein isothiocyanate (QCF) solution. The inoculation and activation of *E. coli* are carried out under aseptic conditions. Tools such as culture medium, Petri dishes, Erlenmeyer flasks, pipettes, pipette tips, etc., need to be autoclaved. The sample solution penetrates from the Oxford cup into the solid medium, then the antibacterial ingredient of the sample decreases with decreasing the concentration of diffusion, creating a concentration gradient at the medium. The multiplication of the bacteria is hindered where the antimicrobial component works, followed by the appearance of highly visible circles known as inhibition circles. The diameter of the inhibition circle reflects the strength of the antibacterial ability of the sample. The culture of *E. coli* is shown in Figure 7, and the criteria for determining the degree of antibacterial sensitivity in the experiment are shown in Table 2 [44,45,46]. The diameter data and sensitivity of the inhibition zone are shown in Table 3. The medium of No. 1 is NC, and there is no bacterial growth in the medium, which proves that the entire experimental operation is aseptic. The medium of No. 2 is covered with *E. coli* and there is no inhibition zone. The mediums of No. 3, 4, 5, 6, 7, 8 are full of *E. coli* and the diameters of the inhibition zone are 12 mm, 14 mm, 12 mm, 8 mm, 12 mm, and 17 mm. The sensitivity of the samples to *E. coli* are moderately sensitive, moderately sensitive, moderately sensitive, not sensitive, moderately sensitive, and highly sensitive, respectively. The PC proved that the culture medium has no other bacteria growth.

**Figure 8:**
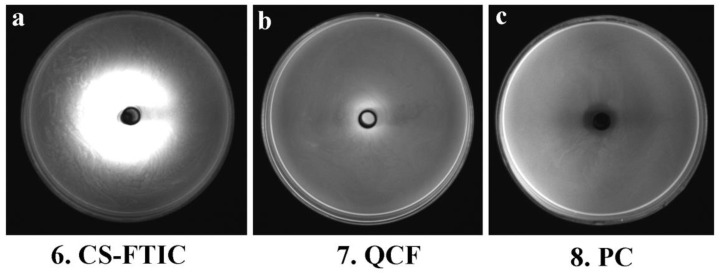
Gel system imaging pictures of CS-FTIC (**a**), QCF (**b**) and PC (**c**).

Figure 8 shows the images of the medium of No. 6, 7 and 8 under the gel imaging system. The medium without fluorescein (No. 8) has no fluorescence under blue light excitation; the sample of No. 6 medium has a widespread range, and the fluorescence aperture diameter in the gel imaging system is large, but the inhibition zone and the fluorescence aperture are not matched, thus the antibacterial performance is poor; the fluorescence aperture of No. 7 medium in the gel imaging system matches the antibacterial circle so that the antibacterial performance of QCF can be visualized through the gel imaging system. Here, comparing the work of other chitosan-based drug-loaded particles, the advantages of our work are reflected (Table 4).

## 4. Conclusions

In summary, chitosan–quercetin (CS-QT) drug-loaded nanoparticles have been successfully labeled by FITC. The SEM and FTIR images demonstrate the successful cross-linking of fluorescein with chitosan and quercetin, and TEM image indicates that the diameter of QCF is about 500 nm. Furthermore, the effect of drug-loading efficiency, encapsulation efficiency, and antioxidant properties have been discussed. When the volume ratio of chitosan (2 mg/mL) to quercetin (1 mg/mL) is 5:1, the drug-loaded rate of the sample reaches 8.39%, and the encapsulation rate reaches 83.65% and exhibits good antioxidant capacity. In our experiments, FITC is used for labeling, which makes the experimental results visualized. The sample has an antibacterial effect on *E. coli*, and the fluorescence aperture of the sample is consistent with the antibacterial circle, so the antibacterial performance of the sample can be visualized. The fluorescent-labeled nanomedicine prepared simply in this experiment exhibits antibacterial properties, which provides a strategy for observing the release and action of the drugs. The design of this novel encapsulation structure based on chitosan nanoparticles will facilitate the development of novel smart drug-loading materials with potential applications in chemotherapy and physical therapy.

## Figures and Tables

**Table 1:** Drug-loaded rate and encapsulation rate of different ratios of samples.

Volume Ratio	pH	Ab	DL (%)	EE (%)
1:1	6.20	3.33	6.79	73.1
2:1	5.78	1.49	7.26	81.9
3:1	5.65	1.06	7.92	82.3
4:1	5.46	0.629	8.15	82.4
5:1	5.45	0.618	8.39	83.7
6:1	5.43	0.555	8.04	82.4
7:1	5.40	0.527	7.89	82.6
8:1	5.37	0.502	7.01	82.1

**Table 2:** Criteria for judging antimicrobial sensitivity.

Diameter o Bacteriostatic Circle (mm)	Experimental Result
≤8	not sensitive
8 < d < 10	low sensitivity
10 ≤ d < 15	moderately sensitive
15 ≤ d < 20	highly sensitive
≥20	extremely sensitive

**Table 3:** Antibacterial sensitivity analysis table.

Sample	Diameter of Bacteriostatic Circle (mm)	Sensitivity
1	8	not sensitive
2	8	not sensitive
3	12	moderately sensitive
4	14	moderately sensitive
5	12	moderately sensitive
6	8	not sensitive
7	12	moderately sensitive
8	17	highly sensitive

**Table 4:** Comparison of fluorescent probe technology with other previously reported work.

Loaded Nanoparticles	Fluorescent Probes	Bacteria	Ref.
Chitosan-quercetin	fluorescein isothiocyanate	*E. coli*	This work
polycaprolactone/quaternized chitosan	-	*E. coli*	[47]
chitosan/pectin-based silver nanoparticle films	-	*E. coli*	[48]
CS–Cu^2+^ nanoparticle	-	*E. coli, Staphylococcus aureus, Candida albicans*	[49]
chitosan/protamine hybrid nanoparticles	-	*E. coli*	[50]
chitosan–lysozyme nanoparticles	-	*E. coli*	[51]
Curcumin-loaded Chitosan Tripolyphosphate Nanoparticles	-	*Staphylococcus aureus*	[52]

## Data Availability

The datasets generated during and/or analyzed during the current study are available from the corresponding author on reasonable request.
